# The Applied Development of a Tiered Multilocus Sequence Typing (MLST) Scheme for *Dichelobacter nodosus*

**DOI:** 10.3389/fmicb.2018.00551

**Published:** 2018-03-23

**Authors:** Adam M. Blanchard, Keith A. Jolley, Martin C. J. Maiden, Tracey J. Coffey, Grazieli Maboni, Ceri E. Staley, Nicola J. Bollard, Andrew Warry, Richard D. Emes, Peers L. Davies, Sabine Tötemeyer

**Affiliations:** ^1^School of Veterinary Medicine and Science, University of Nottingham, Nottingham, United Kingdom; ^2^Department of Zoology, University of Oxford, Oxford, United Kingdom; ^3^Advanced Data Analysis Centre, University of Nottingham, Nottingham, United Kingdom

**Keywords:** footrot, *Dichelobacter nodosus*, MLST genotyping, core genome multilocus sequence typing, whole genome multilocus sequence typing (wgMLST), cgMLST

## Abstract

*Dichelobacter nodosus* (*D. nodosus*) is the causative pathogen of ovine footrot, a disease that has a significant welfare and financial impact on the global sheep industry. Previous studies into the phylogenetics of *D. nodosus* have focused on Australia and Scandinavia, meaning the current diversity in the United Kingdom (U.K.) population and its relationship globally, is poorly understood. Numerous epidemiological methods are available for bacterial typing; however, few account for whole genome diversity or provide the opportunity for future application of new computational techniques. Multilocus sequence typing (MLST) measures nucleotide variations within several loci with slow accumulation of variation to enable the designation of allele numbers to determine a sequence type. The usage of whole genome sequence data enables the application of MLST, but also core and whole genome MLST for higher levels of strain discrimination with a negligible increase in experimental cost. An MLST database was developed alongside a seven loci scheme using publically available whole genome data from the sequence read archive. Sequence type designation and strain discrimination was compared to previously published data to ensure reproducibility. Multiple *D. nodosus* isolates from U.K. farms were directly compared to populations from other countries. The U.K. isolates define new clades within the global population of *D. nodosus* and predominantly consist of serogroups A, B and H, however serogroups C, D, E, and I were also found. The scheme is publically available at https://pubmlst.org/dnodosus/.

## Introduction

Ovine footrot is the leading cause of lameness and a significant welfare issue in the sheep industry (Goddard et al., 2006). The disease results in severe lameness, which has a large financial impact on farmers, surmounting to an estimated cost of £24 million per year in the U.K. alone (Nieuwhof and Bishop, 2005). Recent estimates suggests that 8–10% of sheep within a single flock are affected by footrot within the U.K. (Wassink et al., 2003). Footrot characterized by the separation of the hoof horn from the underlying tissue, is caused by the weakly pathogenic bacterium *Dichelobacter nodosus* (*D. nodosus*) (Egerton et al., 1969). While its role has long been well defined, control and potential eradication is still unachievable in the U.K.

The understanding of the global distribution is that *D. nodosus* can be split into two genetic clades. Clade one comprises of strains isolated from footrot with underrunning or “virulent” lesions and clade two which contains isolates from non-underrunning or “benign” footrot infections (Kennan et al., 2014). These clades are defined by a single amino acid change within the extracellular protease coding region, creating an acidic protease isoenzyme 2 (*aprV2*) virulent strain and a basic protease isoenzyme 2 (*aprB2*) benign strain (Kennan et al., 2010).

The current epidemiological understanding of *D. nodosus* shows it is ubiquitous within the global sheep population (Kennan et al., 2014), with relatively new presence confirmed in Brazil (Aguiar et al., 2011) and Scandinavia (Gilhuus et al., 2013; Frosth et al., 2015). There are multiple molecular typing methods available for *D. nodosus* to assist in the clustering and generation of distribution patterns. Pulse Field Gel Electrophoresis (PGFE) (Zakaria et al., 1998), often described as the “gold standard” of lab based molecular typing (Arbeit et al., 1990) was quickly followed by a Restriction Fragment Length Polymorphism (RFLP) method (Ghimire and Egerton, 1999). However, both are time consuming and require a large amount of input DNA and suffer from inter-lab biases. Infrequent restriction site PCR has also been implemented but was shown to be less robust than the RFLP (Buller et al., 2010). More recently Multiple Locus Variable Number Tandem repeat (VNTR) analysis has proven to be a reproducible and high discriminatory method (Russell et al., 2014), however further analysis is limited due to VNTR only using PCR and gel electrophoresis visualization to draw conclusions.

The typing method that had yet to be applied to the epidemiological surveillance and study of *D. nodosu*s is multilocus sequence typing (MLST). MLST uses DNA sequence variation, in a set of genes required for basic cellular maintenance, to define allelic differences (Maiden et al., 1998), and has become the gold standard for population analyses of bacterial pathogens. Although the number of MLST loci selected differs between species, seven or eight loci are routinely used, with extended MLST schemes utilizing up to 10 (Jolley and Maiden, 2014). The main benefits of MLST are that it is portable, not suffering from inter-lab variation and highly reproducible. The analysis is also automated through server based databases (Jolley and Maiden, 2010) removing the bias potentially incorporated through different users and the complexity of data analysis. Sequence types are created by the generation of an allelic profile. This is a series of numbers based on novel sequence variation present in the seven alleles (Jolley and Maiden, 2014), for example the first loci combination for isolate one will be designated the starting profile (1-1-1-1-1-1-1). Any subsequent variation within an allele will generate allele two (e.g., 1-1-2-1-1-1-1) and so on for all unique loci for each isolate.

Since the genomics era and the associated reduction in cost of high throughput sequencing, MLST has been expanded to enable it to stay a relevant and useful tool. The cost of sequencing several MLST loci is now becoming comparable to whole genome sequencing (WGS) prices but the amount of information gathered is magnitudes larger. From the WGS dataset and incorporating core genome MLST (cgMLST) and whole genome MLST (wgMLST) schemes allows for greater differentiation between isolates, however these data are still compatible with established MLST schemes as the loci can be automatically identified from the WGS and compared with standard MLST data (Jolley and Maiden, 2014).

With numerous *D. nodosus* isolates publically available in the NCBI sequence read archive (SRA ID: ERP005873) (Kennan et al., 2014) the development of a tiered MLST scheme was undertaken and applied to further our understanding of the local and global population dynamics of *D. nodosus*, with the aim of developing a robust typing method to define STs, which would be accessible to all, regardless of budget or experience. As there was also a distinct lack of genome sequences available from *D. nodosus* in the U.K., where footrot is endemic, 2,126 swabs were collected from multiple farms, to allow for a comparison to the global strains.

## Methods

### Isolation of U.K. strains from ovine interdigital swabs

Samples (*n* = 2,126) were collected from 10 sheep farms situated within Nottingham, Derby and Northampton from animals with varying disease states. Sterile nylon flock swabs (E-swabs 480CE, Copan U.S.A.) were used for the collection of samples from the interdigital space of sheep and stored in liquid Amies solution at 5°C overnight. The swabs were inoculated onto Hoof Agar plates containing 4% w/v Bacto Eugon agar (BD, U.S.A.), 0.5% w/v Difco Yeast Extract (BD, U.S.A.), 1.5% w/v BBL Beef Extract (BD, U.S.A.), 1% Sodium Chloride and 6.6% w/v ovine hoof powder (Parker et al., 2005) and incubated anaerobically at 37°C. After 7 days plates were visually checked for putative *D. nodosus* and if present sub-cultured onto reduced agar (2%), hoof agar plates and incubated anaerobically at 37°C. Pure colonies were collected from plates in sterile PBS, washed by centrifugation and resuspended in molecular biology grade water (ThermoFisher, U.K.).

### DNA isolation and sequencing

DNA was isolated using the Qiagen Cador Pathogen Mini Kit, following the manufacturers guidelines and eluted in 60 μl of elution buffer. The DNA samples were quantified using the Qubit 3.0 and dsDNA high sensitivity dye (Qiagen). Quantified DNA was sent to MicrobesNG (Birmingham University, U.K.), prepared for sequencing using the Illumina Nextera XT library preparation kit and sequenced on an Illumina MiSeq at 2 × 250 bp [Raw data is available in the Short Read Archive (PRJNA386733)].

### Analysis of sequence data

Sequence reads for publically available isolates collected from Scandinavian, Australian and Indian flocks were downloaded from NCBI SRA (ID: ERP005873) (Kennan et al., 2014). Raw reads were assembled using the A5 and A5-MiSeq pipelines, depending on read length (Tritt et al., 2012; Coil et al., 2015). Briefly, raw reads were analyzed for sequence adaptors using trimmomatic (Bolger et al., 2014) and clipped if necessary, the reads were then error corrected using the SGA *k-mer* based approach (Simpson and Durbin, 2012). Clipped and corrected paired and unpaired reads were assembled using IDBA-UD (Peng et al., 2012) to create rough contigs. These were then scaffolded and extended using SSPACE (Boetzer et al., 2011) before having the clipped and corrected reads realigned using BWA (Li and Durbin, 2009). The scaffolds were then checked for discordant reads indicative of misassembles and scaffolded again using SSPACE (Boetzer et al., 2011).

### Determination of MLST suitable loci

All assembly contig files were uploaded to the *D. nodosus* PubMLST BIGSdb database (https://pubmlst.org/dnodosus/) (Jolley and Maiden, 2010) for analysis. Isolate definitions and metadata were used from the sequence read archive (Kennan et al., 2014; Jackson et al., 2015). To identify the loci suitable for MLST, the annotated assembled genomes were used to identify the core genome using BIGSdb (Jolley and Maiden, 2010). Loci present in ≥95% of isolates were checked for average length and those suitable for standard PCR and Sanger sequencing (500–600 bp) were selected. The locations of these final loci were checked using the reference genome VCS1703A (Accession GCF_000015345.1) to select loci which have a good distribution throughout the genome. Evolutionary rate of loci were assessed by calculation of *d*N*/d*S ratios using start2 (Jolley et al., 2001), with additional calculations of Pairwise Homoplasy Index (PHI) (normal and permutation based), Max Chi^2^ and Neighbor similarity score using PHIPack (Bruen et al., 2006) to test for levels of recombination. Contig files were scanned and allele numbers were defined using BIGSdb (Jolley and Maiden, 2010). For the initial 107 isolates available, allele numbers for seven of the nine loci were chosen at random, for all possible permutations, to create a unique seven-digit code for each isolate using R (Ihaka and Gentleman, 1996) (Script available at https://github.com/ADAC-UoN/MLST). The numbers were assessed for unique occurrences to determine pseudo-sequence types.

### Primer design

Loci coding sequences were extracted from the contig files using BIGSdb incorporating flanking regions. Consensus sequences were generated to facilitate primer design. Sequences were loaded into NCBI Primer BLAST (https://www.ncbi.nlm.nih.gov/tools/primer-blast/) with settings to ensure the whole coding sequence would be amplified. All primer pairs where chosen for compatible annealing temperatures to allow for all reactions to use the same amplification conditions (**Table 3**). All PCR amplifications were conducted in a 50 μl reaction volume using 50 ng of purified *D. nodosus* chromosomal DNA as template. All reactions were assembled on ice using a final concentration of 200 μM dNTPs, 0.5 μM of forward primer and 0.5 μM or reverse primer (Table 3), 0.02 U/μl of NEB Q5 High-Fidelity DNA Polymerase (New England Biolabs Inc., U.S.A.) in 1x Q5 reaction buffer. A preheated Thermocycler was used with an initial denature of 98°C for 30 s followed by 35 cycles of 98°C for 10 s, 60°C for 25 s, and 72°C for 30 s. A final extension of 2 min at 72°C was used before a hold of 10°C.

### Identification and visualization of the core genome

The genome comparator plugin for BIGSdb was used to determine which loci were shared within the isolates. Those loci identified as core genome and present in ≥95% of the isolates (equating to 715 loci, 53% of loci present in all isolates) were used to develop the cgMLST scheme. Multiple sequence alignments of these loci, using MUSCLE (Edgar, 2004), were performed with BIGSdb and phylogeny was inferred using maximum-likelihood implemented with FastTree, compiled with the Double Precision, to resolve branch lengths of closely related isolates (Price et al., 2010).

### Serogroup and phenotype determination

The assembled contig files were used as the input for IPCRESS (Slater and Birney, 2005) part of the exonerate pipeline. Serogroup determination was completed using the PCR primers developed by (Zhou et al., 2001). The phenotypic (*aprV2/aprB2*) determination made use of the PCR primers created by (Frosth et al., 2012).

## Results

### Selection of MLST loci

The assemblies were assessed for overall quality showing an average of 33 contigs per isolate with 74% of their respective reads passing error correction. There was a median coverage of 330x and 97% of reads for each assembly having a phred score of >40. The core genome analysis identified 1,171 coding sequences that were shared between ≥95% of the isolates. These were filtered for length appropriate for standard PCR leaving 240 coding sequences 500–600 bp in length, 19 of which had confirmed gene identifiers. The preliminary selection of 12 loci (Table 1) was chosen due to their distribution throughout the reference genome (VCS1703A, Accession GCF_000015345.1). The random permutation showed whichever selection of loci was chosen the number of STs did not alter considerably (Median 88, Range 78–92). The 12 loci were assessed for recombination using PHIPack (Bruen et al., 2006) and diversity using START2 (Jolley et al., 2001; Table 2). All loci showed similar levels of recombination and diversity according to Pairwise Nucleotide Diversity (%), NSS, Max Chi (Nieuwhof and Bishop, 2005), Phi Perm, Phi Norm and the *dS/dN* ratio. The *dN/dS* ratios were also all below one, suggesting the polymorphisms are not a result of positive selection pressure and therefore these loci are suitable for the use in the MLST analysis. The reading frames of the loci were examined to ensure they were consistent, however three loci *ompA, purE, rdgB* were all positioned on the reverse strand and therefore were disregarded. The remaining nine loci were used for PCR primer design (Table 3). Due to the difficulties designing primers to amplify the whole locus, *greA* and *purE* were dismissed. The final chosen loci (*dtdA, folk, rlmH, rplI, tsaE, dcd, recR*) were amplified producing clear specific products suitable for sanger sequencing.

**Table 1:** Description of first selection loci.

**Loci**	**Function**	**GC (%)**	**Size (bp)**
**dtdA**	**D-aminoacyl-tRNA deacylase**	**45.6**	**438**
**folk**	**7, 8-dihydro-6-hydroxymethylpterin-pyrophosphokinase**	**47.4**	**495**
ompA	cell envelope biogenesis protein	44.2	585
purE	N5-carboxyaminoimidazole ribonucleotide mutase	49.9	531
rdgB	non-canonical purine NTP pyrophosphatase	47.2	591
rimN	threonylcarbamoyladenosine biosynthesis protein	49.3	573
**rlmH**	**ribosomal RNA large subunit methyltransferase H**	**46.7**	**471**
**rplI**	**50S ribosomal protein L9**	**43.7**	**450**
**tsaE**	**threonylcarbamoyladenosine biosynthesis protein**	**43.7**	**414**
**Dcd**	**Deoxycytidine triphosphate deaminase**	**43.9**	**567**
greA	Transcription elongation factor	42.6	483
**recR**	**DNA recombination protein**	**47.6**	**606**

*Emboldened loci are those used in the final scheme*.

**Table 2:** Metrics associated with first selection loci for MLST scheme.

**Loci**	**Pairwise Nucleotide Diversity (%)**	**NSS**	**Max Chi^2^**	**Phi Perm**	**Phi Norm**	***d*S/*d*N ratio**
***dtdA***	**0.9**	**0.359**	**0.014**	**0.6380**	**0.44400**	**0.0985**
***folK***	**0.8**	**0.005**	**0.556**	**0.0010**	**0.00077**	**0.0498**
*ompA*	1	0.001	0.187	0.3020	0.19500	0.0942
*purE*	0.7	0.109	0.962	0.5030	0.36400	0.0535
*rdgB*	0.6	0.565	0.070	0.5670	0.37800	0.2253
*rimN*	1.1	0.007	0.956	0.0010	0.00001	0.0574
***rlmH***	**0.6**	**0.165**	**0.826**	**0.8290**	**0.66400**	**0.0802**
***rplI***	**1**	**0.010**	**0.898**	**0.1400**	**0.07690**	**0.0707**
***tsaE***	**1.2**	**0.001**	**0.386**	**0.0010**	**0.00029**	**0.3052**
***dcd***	**0.7**	**0.170**	**0.755**	**0.3320**	**0.22800**	**0.0096**
*greA*	0.5	1.000	0.981	1.0000	1.00000	0.1207
***recR***	**0.9**	**0.043**	**0.305**	**0.0210**	**0.00652**	**0.0718**

*Emboldened loci are those used in the final scheme*.

**Table 3:** PCR primers for final chosen MLST loci chosen for the MLST scheme.

**Loci**	**Primer 1 (5′-3′)**	**Primer 2 (5′-3′)**	**Tm°C**
*dtdA*	CGGTCAAAATTGAACAAGGAACA	GATTCAGCGACGTTTGCTCG	60
*folK*	ATACGAAACCGCGCAAATCG	GGTGCGTCCGTTATTGACGA	60
*rlmH*	TTGCTGCCGTTATTCCGACT	CAACCAGCTGCTTTTCGATCC	60
*rplI*	CATTTGCCGGAATCGCTGTA	CAATTAGCAACCGCCGTCAA	60
*tsaE*	TTATCGTTTCGGCAACGCAC	ACCGCACAACAACACATCAC	60
*dcd*	CACGTTGGCTATCGTTTGGC	ATCGCGTTCTGTTCTGGTCT	60
*recR*	AACGCAACGAGAAATGAGCG	TAAAAACATCGCGCGGCAAA	60

### Population of the database

Serogroups had already been assigned to all the existing isolates available in the Shot Read Archive (ID: ERP005873) (Kennan et al., 2014). These were used to assess the efficacy of the PCR primers (Zhou and Hickford, 2001) to be used for the *in silico* designation (Supplementary Table 1). The results from the IPCRESS *in silico* PCR (Slater and Birney, 2005) matched the previous *in vivo* serogroup designation and were therefore used to classify all the newly isolated strains. The same IPCRESS methodology was utilized to identify the protease phenotype based on the primers developed by Frosth et al. (2012).

### Application of the MLST scheme

With the addition of 67 new isolates from this study, the MLST database was comprised of 171 isolate records, with 68 from the U.K., 39 from Australia, 36 from Norway, 17 from Sweden, 8 from Denmark and one each from India, Nepal and Bhutan. The final combination of 7 loci (*dcd, dtdA, folk, recR, rlmH, rplI, and tsaE*) defined between 15 (*tsaE*) and 24 (*folk*) alleles with the average number per loci being 21, this would allow to potentially distinguish between 1.80 × 10 (Frosth et al., 2015) different isolates. The proportion of nucleotide variation of the selected loci ranged from 2.2% (*dcd*) to 4.1% (*tsaE*).

### Genotypic relationship determination using BURST and goeBURST

**Figure 1:**
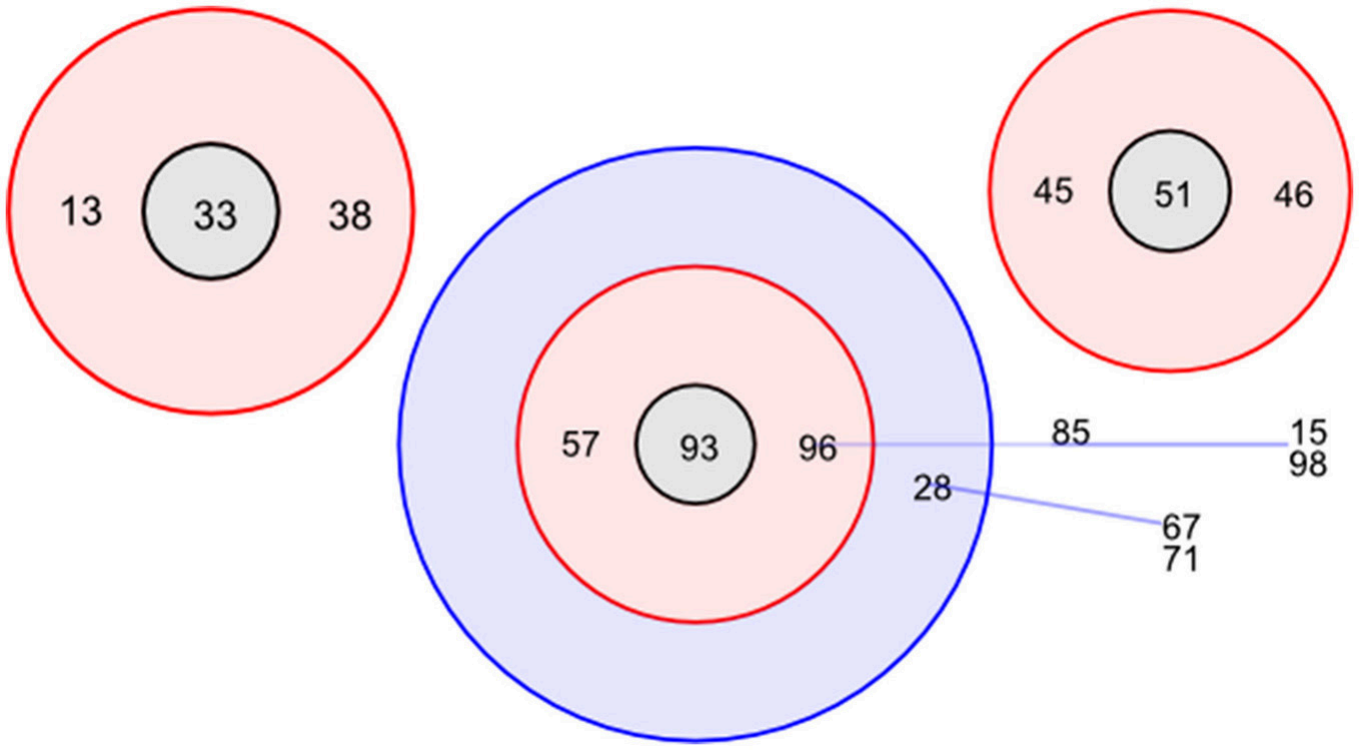
Designation of clonal complexes. Analyses of clonal complexes using BURST (Feil et al., 2004) based on two shared loci (Feil et al., 2004). There was one clonal group determined, encompassing two clonal complexes; ST96 (founder complex) and ST33 without any DLV. Relationships between clonal complex DLV and satellites are identified using colored lines. Red identifies SLV status and blue identifies DLV status.

To determine the lineage of the STs, BURST analysis (Feil et al., 2004) was used based on six shared loci and a bootstrap of 1,000 repetitions. A total of 14 groups were identified, comprising of 3 clonal complexes (Figure 1). ST93 is classed as the founding complex with ST57 and ST96 as single locus variants (SLV), ST28 as double locus variants (DLV) with ST15, ST85, and ST98 being satellites of ST96 and ST67 and ST71 being satellites of ST28. The additional main clonal complexes are based on ST33 as the second founder with ST13 and ST38 as SLV and ST51 as the third founder with ST45 and ST46 as SLV (Figure 1). Interestingly there are 46 singletons within the 115 STs.

**Figure 2:**
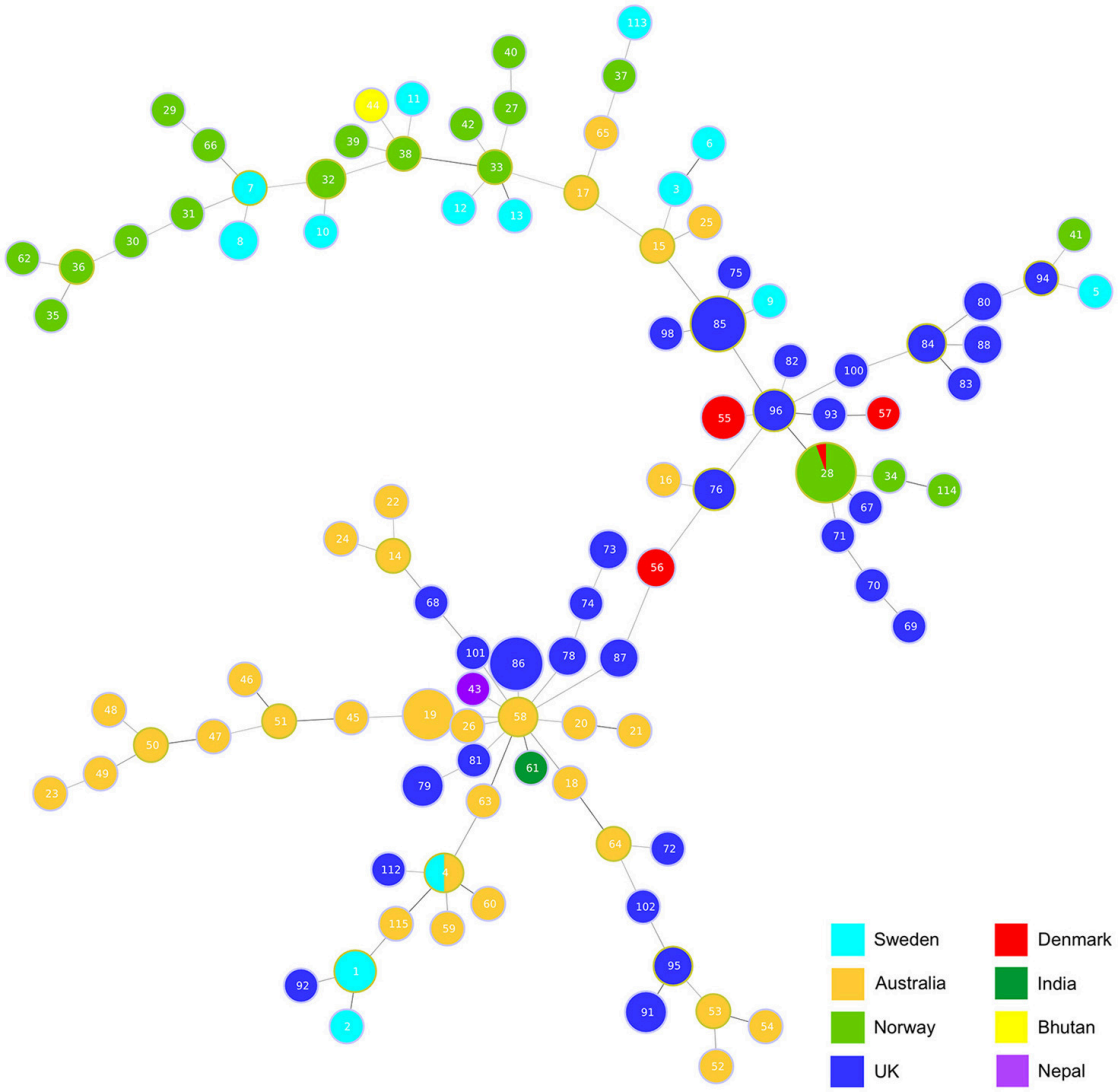
Hypothetical genotypic relatedness of clonal complexes. Analysis of relationships between clonal complexes based on two shared loci using goeBURST (Francisco et al., 2009). The size of each circle represents how prevalent the ST is within the database.

Analysis using goeBURST (Global optimized eBURST) (Francisco et al., 2009), an adaptation of BURST, identified the relatedness of the clonal complexes (Figure 2). The analysis highlights an Australian isolate ST58 seems to be the main group founder with the most SLVs. This ST incorporates some of the oldest isolates VCS1006 and VCS1008 from 1974, and is closely linked to the first isolate of *D. nododus* VPI2340 (ST4) which is a ST also found in Sweden.

### Core genome and whole genome MLST

**Figure 3:**
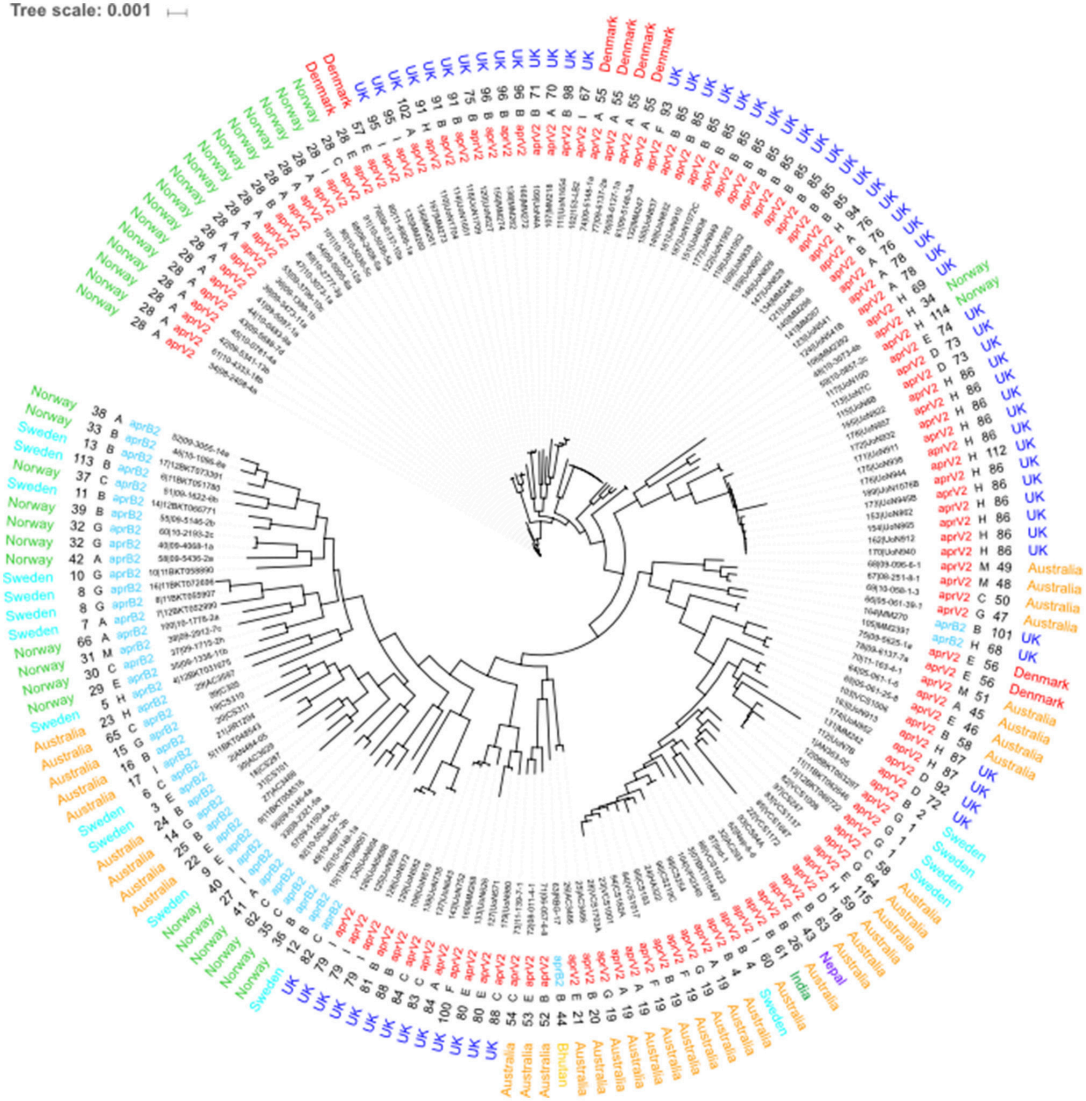
Core genome MLST of *D. nodosus*. Phylogeny inferred using maximum-likelihood, implemented in FastTree (Price et al., 2010). Labels from leaf tips outwards are Isolate database identification number and name, phenotype, sequence type, serogroup and country of origin. Serogroup and phenotype were determined using IPCRESS *in silico* PCR (Slater and Birney, 2005) using primers developed by Zhou and Hickford (2001) and the *aprV2/B2* qPCR protocol developed by Frosth et al. (2012).

**Figure 4:**
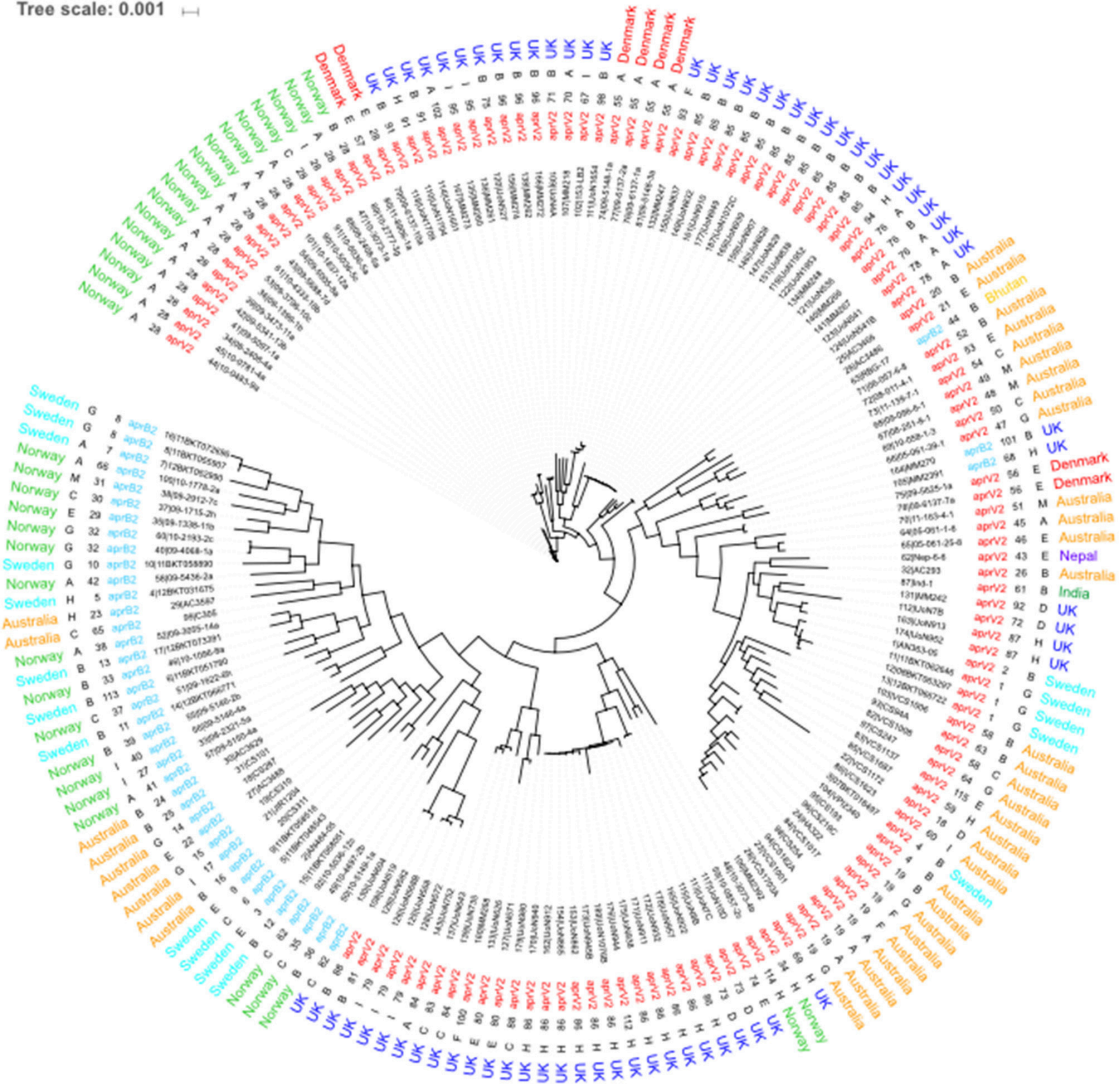
Whole genome MLST of *D. nodosus*. Phylogeny inferred using maximum-likelihood, implemented in FastTree (Price et al., 2010). Labels from leaf tips outwards are Isolate database identification number and name, phenotype, sequence type, serogroup and country of origin. serogroup and phenotype were determined using IPCRESS *in silico* PCR (Slater and Birney, 2005) using primers developed by Zhou and Hickford (2001) and the *aprV2/B2* qPCR protocol developed by Frosth et al. (2012).

The 115 STs identified from the 171 isolates had 5 major STs with ST28 (*n* = 18), ST85 (*n* = 12), ST86 (*n* = 11) and ST19 (*n* = 8) being the most frequent (Figure 3). STs seemed to correlate mostly to country of origin, apart from ST28 which encompasses Norwegian and Danish isolates and ST4 which is present in both Australian and Swedish isolates. Overall, 79% of all the 171 isolates in the database were designated as *aprV2* positive. The determination of serogroups from these data within the population shows a higher incidence of serogroups A (*n* = 35–19.88%), B (*n* = 53–20.9%) and H (*n* = 23–13.45%), with serogroups C (*n* = 14–8.19%), E (*n* = 16–9.36%), G (*n* = 14–8.19%), and I (*n* = 13–6.43%) less frequent and groups D (*n* = 5–2.92%), F (*n* = 4–2.34%), and M (*n* = 4–2.34%) being the least frequent. The greater complement of genes used in the core genome phylogeny determination afforded enhanced resolution on the relationships between isolates (Figure 3). The main U.K. STs (ST85 and ST86), clustered together, and also incorporated STs ST34 and ST114 from Norway and ST55 from Denmark and two additional STs from the U.K. (ST101 and ST68). The main advantage of using cgMLST is to enable a higher resolution of discrimination between the isolates. This is shown by the length of the nodes, which relate to the evolution process because of the base changes acquired. The increase of the number of loci analyzed for the wgMLST only shows some minor alterations to node length and a few rearrangements within the clades (Figure 4).

## Discussion

Strain typing techniques allow for the tracking of acquired genetic alteration, a powerful epidemiological tool most often used in disease outbreaks. Genetic epidemiology has long been used to track and infer relatedness within and between species of bacteria. Various tools have been applied to *D. nodosus*; RFLP (Ghimire and Egerton, 1999), infrequent restriction site PCR (Zakaria et al., 1998) and the traditional “Gold Standard” epidemiological tool PGFE (Buller et al., 2010), to try and understand the diversity and inform approaches to combat footrot. However, these often suffer with poor reproducibility between laboratories and are time consuming and expensive. Multiple locus VNTR (Russell et al., 2014) made some progress in providing a cost effective and portable tool, however in the age of genomics, there is a limit to its use in future research as its being based purely on PCR product size. MLST is a highly reproducible, unambiguous, portable and well established method. The inclusion of wgMLST and cgMLST allows for greater clonal differentiation, without any of the limitations of PGFE, establishing itself as the new “gold Standard” for epidemiological investigation. The metabolic diversity of bacteria has prevented the development of a universal MLST scheme, however there are currently 142 MLST schemes held at the University of Oxford, The University of Warwick and the Pasteur Institute, France (https://pubmlst.org/databases.shtml). MLST can be expensive, but utilizing whole genome sequencing is scalable to incorporate core genome typing and whole genome typing, which provides some “future proofing” of the technique (Jolley and Maiden, 2014).

Currently the *D. nodosus* database contains 171 isolates with 115 STs (September 2017), suggesting a high level of diversity with a low level of recombination which is reflected in the grouping of isolates and branch lengths shown in the cgMLST and wgMLST analyses. The movement away from the traditional definitions of virulent and benign isolates is a reflection of our greater understanding of *D. nodosus* and its ability to cause disease. Both phenotypes have been isolated during the sample collection in this study from cases of underrunning of the hoof horn. A more robust definition is based on the allele type (*aprV2* or *aprB2*). The application of the *in-silico* serogroup determination also adds to the cost saving and additional value of WGS. The determination of serogroups from these data within the U.K. population shows a higher incidence of serogroups B (35.29%) and H (26.47%) which matches the most prevalent serogroups discovered in previous studies (Moore et al., 2005). However, the proportion of each has shifted, with serogroup B now being the most commonly isolated instead of serogroup H. This suggests that while the overall population is stable and has maintained some consistency over the last 10 years, the prevalence of serogroup B seems be on the increase.

The determination of ST seems to be independent of serogroup and phenotype, suggesting selective pressure on the MLST loci is not linked to either the *fimA* or *aprV2/B2* loci. However, STs are related to the country of origin with very few being identified in multiple countries. These data reinforce the conclusions drawn from the VNTR scheme (Russell et al., 2014) that the global population consists of local sub-populations with limited geographical distribution. Interestingly the U.K. isolates seem to link smaller clusters together from Australia and the Scandinavian regions. Based on core genome analysis, the Indian, Bhutanese and Nepalese isolates seem highly similar to Australian isolates with a rare occurrence of an *aprV2* isolate found in Sweden. The movement of isolates can be inferred due to the relationship between clonal complexes designated by goeBURST and matched with historical accounts of sheep trade.

Whether using VNTR or standard MLST, limiting epidemiological analyses to a small subset of loci can falsely identify relationships which makes the utilization of WGS with core and whole genome analyses even more important for the longevity and usefulness of the dataset. This study has addressed the lack of knowledge on the global relatedness of *D. nodosus* and interestingly highlights the lack of recombination at many levels of analysis. Wider sampling in other regions of the U.K. and in other countries will improve the epidemiological understanding of this economically important species.

## Ethics statement

This study was reviewed and approved by the University of Nottingham, School of Veterinary Medicine and Science ethical review committee ERN: 1144 140506 (Non ASPA).

## Open access data

All sequence data generated for this study is held in the NCBI SRA and EMBL ENA under the accession number PRJNA386733 and scripts used are available at https://github.com/ADAC-UoN/MLST.

## Author contributions

AB created and populated the MLST database and performed all the analysis. AB and ST have written the manuscript. AB, KJ, and TC designed the MLST database. KJ and MM created and hosted Pub MLST and implemented the MLST database. AB, PD, and NB collected the swabs to isolate *D. nodosus* from the U.K. farms. AB and CS processed the swabs from the U.K. farms. CS purified the isolates and prepared the samples for sequencing. GM provided support for the isolation protocol and some additional isolates to sequence. RE and AW provided support for the generation of various scripts to analyse the data. ST, TC, and RE developed the idea of the MLST scheme. All authors have read the manuscript and provided input.

### Conflict of interest statement

The authors declare that the research was conducted in the absence of any commercial or financial relationships that could be construed as a potential conflict of interest.
