# Supplementary material for: The Applied Development of a Tiered Multilocus Sequence Typing (MLST) Scheme for Dichelobacter nodosus
Source: Front Microbiol. 2018 Mar 23;9:551. doi: 10.3389/fmicb.2018.00551 (PMC5876313; doi:10.3389/fmicb.2018.00551)
Supplement: Supplementary file 1 [file Table1.docx]

| Serogroup | Forward | Reverse |
| --- | --- | --- |
| A | CCTTAATCGAACTCATGATTG | AGTTTCGCCTTCATTATATTT |
| B | CCTTAATCGAACTCATGATTG | CGGATCGCCAGCTTCTGTCTT |
| C | CCTTAATCGAACTCATGATTG | AGAAGTGCCTTTGCCGTATTC |
| D | CCTTAATCGAACTCATGATTG | TGCAACAATATTTCCCTCATC |
| E | CCTTAATCGAACTCATGATTG | CACTTTGGTATCGATCAACTTGG |
| F | CCTTAATCGAACTCATGATTG | ACTGATTTCGGCTAGACC |
| G | CCTTAATCGAACTCATGATTG | CTTAGGGGTAAGTCCTGCAAG |
| H | CCTTAATCGAACTCATGATTG | TGAGCAAGACCAAGTAGC |
| M | ATCCCTGCATACAACGACTACAT | CGATGGGTCAGCATCTGGACC |

Primers used for in-silico serogrouping based on Zhou and Hickford (2001)
